# *C. elegans* does a spit take

**DOI:** 10.7554/eLife.71813

**Published:** 2021-08-03

**Authors:** Michael Hendricks

**Affiliations:** Department of Biology, McGill UniversityMontrealCanada

**Keywords:** subcellular muscle contraction, subcellularly localized calcium transients, hourglass circuit motif, motor control, neural circuitry, behavior, *C. elegans*

## Abstract

Eating can turn into spitting because individual parts of a muscle cell are able to contract in different ways.

**Related research article** Sando SR, Bhatla N, Lee ELQ, Horvitz HR. 2021. An hourglass circuit motif transforms a motor program via subcellularly localized muscle calcium signaling and contraction. *eLife* **10**:e59341. doi: 10.7554/eLife.59341

Like all animals, the tiny worm *Caenorhabditis elegans* dislikes bad-tasting food – a probably common occurrence in the compost where it lives (Frézal and Félix, 2015). In fact, recent data showed that just like you and I, the worms can spit out foul-tasting chemicals such as reactive oxygen species (Bhatla and Horvitz, 2015b; Bhatla et al., 2015a). To explain how these types of behaviors can emerge, scientists often focus on correlations between brain activity, sensory inputs and behavioral outputs. However, while neural activity understandably comes to the fore, attempts at mechanistic explanations will always fall short if they do not include another class of excitable cells that are essential for behavior: muscles. Now, in eLife, Robert Horvitz and colleagues from Massachusetts Institute of Technology (MIT) and University of California, Berkeley – including Steven Sando as first author – report on the impressive complexity in muscle coordination required for worms to spit out their food (Sando et al., 2021).

The feeding organ of *C. elegans* contains a pump that ingests and grinds bacteria before passing them on to the gut. Like the nervous system in the guts of mammals, this ‘pharynx’ is somewhat a fiefdom of its own. Formed of 20 neurons and 20 muscle cells isolated from the rest of the nervous system (both physically and in terms of neuronal connections), the organ regulates food intake autonomously (Avery and Shtonda, 2003; White et al., 1986). In particular, two structures in the pharynx ensure that the worms can eat properly: the metastomal filter stops large particles from entering while the pharyngeal valve acts as a one-way check and keeps food moving in the right direction (Fang-Yen et al., 2009). So how can such a dedicated pump suddenly reverse direction?

By analyzing high-speed videos, Sando et al. noticed that when the worms are spitting, the rate of pumping increases in the pharynx. This seems counterintuitive: if food tasted unpleasant, you probably would not start gorging on it even faster. However, the metastomal filter and pharyngeal valve are held open during this increase, allowing the contents of the pharynx to be rapidly flushed back into the environment.

To examine how the valve stayed open during spitting, the team then focused on a set of muscles known as pm3s. These three muscle cells contract and relax rhythmically to help the pharynx pump food, and to allow the pharyngeal valve to open and close. However, during spitting, pm3s play two simultaneous roles: the anterior portions of the cells stay contracted to keep the valve open, while their posterior sections rapidly contract and relax to drive food out of the pharynx.

To confirm that these changes came from pm3s themselves – and not from forces impinging on the muscle or the valve – Sando et al. had a closer look at muscle activation during pumping and spitting. To do so, they expressed a calcium-sensitive fluorescent protein in pharyngeal muscles, as the concentration of calcium ions increases inside a contracting cell. This revealed that in spitting animals, sustained calcium signals were localized around the pharyngeal valve. This result is consistent with the anterior portion of pm3s (and only this portion) contracting to hold the valve open. But how is this complex activity state of pm3 regulated?

A pharyngeal neuron call M1 is essential for spitting – killing this cell with a laser stops the spitting response in worms. Based on the cells that M1 connects to and further experiments, Sando et al. suggest that this neuron integrates multiple signals that correspond to noxious tastes. The signaling output of the M1 neuron varies in strength according to these inputs: weak activation leads to opening of the pharyngeal valve, and only strong activation results in the valve opening and increased pumping necessary to eject food. In turn, various degrees of spitting behavior could emerge from these different inputs thanks to local contraction of cellular portions of the pm3 muscles.

Sando et al. stopped short of exploring the cellular mechanisms that allow local contraction of pm3s. In other systems, like mammalian smooth muscle, contractility patterns are determined by the spatial and temporal dynamics of calcium ions. These patterns arise from a complex interplay between various sources of ions and the channels or regulatory proteins that compartmentalize and shape calcium dynamics inside a cell. A similar mechanism could be happening here, with various levels of M1 activation targeting different sources of – or regulatory pathways for – intracellular calcium ions in pm3s.

Taken together, the results from Sando et al. highlight that muscles are not just passive conduits for neural commands: instead, they can exhibit dynamics that arise from the interplay between neural signals and their own, varying physiological properties. The functional insights of this study, along with the power of *C. elegans* genetics, offers an opportunity to study complex muscle dynamics and their neural regulation in a compact and accessible system.
